# Melatonin and Rapamycin Attenuate Isoflurane-Induced Cognitive Impairment Through Inhibition of Neuroinflammation by Suppressing the mTOR Signaling in the Hippocampus of Aged Mice

**DOI:** 10.3389/fnagi.2019.00314

**Published:** 2019-11-19

**Authors:** Hui Yuan, Guorong Wu, Xiaojie Zhai, Bo Lu, Bo Meng, Junping Chen

**Affiliations:** Department of Anesthesiology, HwaMei Hospital, University of Chinese Academy of Sciences, Ningbo, China

**Keywords:** mTOR, melatonin, neuroinflammation, cognitive impairment, isoflurane

## Abstract

Melatonin exerts neuroprotective effects on isoflurane-induced cognitive impairment. However, the underlying mechanism has yet to be elucidated. The present study sought to determine if melatonin confers its beneficial effects by acting on mammalian target of rapamycin (mTOR) and attenuates the neuroinflammation in the hippocampus of aged mice. A total of 72 male C57BL/6 mice, 16-month-old, were randomly and equally divided into six groups: (1) the control group (CON); (2) the rapamycin group (RAP); (3) the melatonin group (MEL); (4) the isoflurane group (ISO); (5) the rapamycin + isoflurane group (RAP + ISO); and (6) the melatonin + isoflurane group (MEL + ISO). RAP, RAP + ISO, MEL, MEL + ISO groups received 1 mg/kg/day mTOR inhibitor rapamycin solution or 10 mg/kg/day melatonin solution, respectively, intraperitoneally at 5:00 p.m. for 14 days consecutively. Mice in the CON and ISO groups were administered an equivalent volume of saline. Subsequently, ISO, RAP + ISO, and MEL + ISO groups were exposed to inhale 2% isoflurane for 4 h; the CON, RAP, and MEL mice received only the vehicle gas. Then, the memory function and spatial learning of the mice were examined via the Morris water maze (MWM) test. mTOR expression was detected via Western blot, whereas the concentration of inflammatory cytokines tumor necrosis factor (TNF)-α, interleukin (IL)-1β, IL-6 and that of melatonin was quantified with enzyme-linked immunosorbent assay (ELISA). Melatonin and rapamycin significantly ameliorated the isoflurane-induced cognitive impairment and also led to a decrease in the melatonin levels as well as the expression levels of TNF-α, IL-1β, IL-6, and p-mTOR in the hippocampus. In conclusion, these results showed that melatonin and rapamycin attenuates mTOR expression while affecting the downstream proinflammatory cytokines. Thus, these molecular findings could be associated with an improved cognitive function in mice exposed to isoflurane.

## Introduction

Perioperative neurocognitive disorders (PND) is defined as a cognitive impairment after surgery and anesthesia, especially in elderly patients (Evered et al., 2018). PND affects a wide range of cognitive functions, such as learning, memory, attention, and language comprehension, resulting in decreased quality of life and increased mortality in patients (Steinmetz et al., 2009). Patients >65 years of age are at greater risk, and a subset of these patients progress toward dementia within 3–5 years (Kapila et al., 2014). However, the mechanism of PND has not yet been clarified.

Mammalian target of rapamycin (mTOR) is a conserved serine/threonine protein kinase and belongs to the phosphoinositide-3-kinase family. It has been reported to mediate a myriad of intercellular processes, such as energy metabolism, translation, autophagy, and apoptosis (Saxton and Sabatini, 2017). In the nervous system, mTOR plays a vital role in maintaining brain function. Studies have shown that mTOR promotes learning and memory formation through synaptic enhancement that relies on protein synthesis (Hoeffer and Klann, 2010), and the dysregulation of mTOR has resulted in impaired learning, memory, and social behavior in mice (Banko et al., 2007; Ehninger et al., 2008). Several studies have shown that mTOR may be related to neurological and aging-associated diseases, such as Alzheimer’s disease (AD) (Bockaert and Marin, 2015). It has been found that upregulation of mTOR leads to accumulation of highly phosphorylated tau in AD (Li et al., 2005), and the mTOR inhibitor rapamycin enhances spatial learning and memory function (Halloran et al., 2012; Majumder et al., 2012). In addition, recent studies have shown that the overexpression of mTOR in the hippocampus is associated with the development of PND in elderly mice (Zhang C. et al., 2016). Therefore, it has been proposed that rapamycin and other mTOR inhibitors exhibit therapeutic effects against PND (Yang et al., 2013).

Furthermore, neuroinflammation is also a major pathway that leads to PND (Skvarc et al., 2018). Animal studies demonstrated that the pathophysiological process of PND might be related to the inflammatory cytokines TNF-α, IL-1β, and IL-6 in the hippocampus (Tang et al., 2011; Hovens et al., 2014; Wang et al., 2017). The rodent experiments showed that the inhibition of the expression of central proinflammatory cytokine signaling can lower the severity of postoperative memory impairment (Cibelli et al., 2010; Terrando et al., 2010; Barrientos et al., 2012; Jiang et al., 2015). Further studies demonstrated that mTOR expression is vital in facilitating neuroinflammation in many neurodegenerative diseases (Soltani et al., 2018). However, whether mTOR is involved in the hippocampal neuroinflammation during the pathophysiology of PND is yet to be evaluated.

Melatonin is a product of the pineal gland with a role in regulating endogenous circadian rhythms. A recent study also demonstrated neuroprotective effects (Hardeland, 2012). In mammals, the abnormal levels of melatonin in the hippocampus were related to several aging-related diseases (Hardeland, 2012), including AD and Parkinson’s disease (PD). These conditions also depicted the altered expression of melatonin receptor (Sanchez-Hidalgo et al., 2009). In addition, the symptoms of these diseases can be significantly improved when exogenous melatonin is utilized (McKenna et al., 2012; Pandi-Perumal et al., 2012).

In recent years, several studies reported that melatonin has a functional role in improving cognitive function (Jansen et al., 2006; Furio et al., 2007). Electrophysiological studies have shown that this effect is produced by melatonin in the hippocampus (Liu X.J. et al., 2013; Zhang S. et al., 2016). A recent study showed that the level of melatonin in the hippocampus could be increased by the intraperitoneal injection of exogenous melatonin, thereby reducing the degree of cognitive dysfunction (Liu et al., 2017). However, the molecular mechanism of this effect is not yet clarified. Therefore, we proposed that melatonin might function in mTOR signaling pathways and ameliorate the postoperative cognitive dysfunction.

In the present study, a mouse model was employed to determine whether mTOR participates in the pathophysiology of PND by aggravating the hippocampal neuroinflammation and whether melatonin ameliorates the postoperative cognitive impairment by inhibiting the expression of mTOR in the hippocampus.

## Materials and Methods

### Animals

All animals were obtained from the Experimental Animal Center of Zhejiang Academy of Medical Sciences, China. All animal protocols were approved by the Ningbo University Laboratory Animal Center under permit number No. SCXK (ZHE 2014-0001) and conducted in strict compliance with the Guide for the Care and Use of Laboratory Animals (National Institutes of Health, United States). A group of 16-month-old healthy male C57BL/6 mice was maintained at 12-h light/dark cycle at 20–25°C and 60–70% humidity. Water and food were freely available for at least 1 day before treatment or isoflurane exposure.

### Experimental Groups and Drug Treatment

**FIGURE 1:**
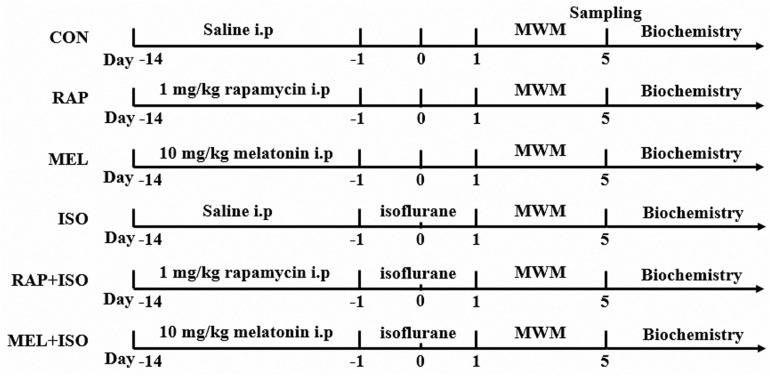
The schematic diagram of the experimental procedures.

A total of 72 mice were divided randomly into six groups (*n* = 12): (1) the control group (CON); (2) the rapamycin group (RAP); (3) the melatonin group (MEL); (4) the isoflurane group (ISO); (5) the rapamycin + isoflurane group (RAP + ISO); (6) the melatonin + isoflurane group (MEL + ISO). Mice in RAP and RAP + ISO groups were administered mTOR inhibitor rapamycin solution at a dosage of 1 mg/kg/day at 5:00 p.m., intraperitoneally, for 14 consecutive days as described previously (Fu et al., 2017). Mice in MEL and MEL + ISO groups were administered melatonin solution at 10 mg/kg/day at 5:00 p.m., intraperitoneally, for 14 consecutive days as described previously. Melatonin was first dissolved in absolute ethanol and then diluted with 0.9% saline (Liu Y. et al., 2013). CON and ISO were administered equivalent volumes of vehicle (normal saline) in the same way. On day 15, mice in ISO, RAP + ISO, MEL + ISO groups were exposed to isoflurane, but the CON, RAP, and MEL mice received only the vehicle gas. The experimental protocol is illustrated in Figure 1.

### Isoflurane Exposure

Mice were administered isoflurane as described previously (Ni et al., 2013). Briefly, mice were first placed in an anesthetic chamber, and parameters such as the rectal temperature, arterial oxygen saturation, heart rate, and blood pressure were monitored. Then, the animals were exposed to 4 h inhalation of 2% isoflurane. The concentration of isoflurane, carbon dioxide was continuously monitored by a gas monitor. At the end of isoflurane exposure, 100% oxygen was administered until the mice can turn itself from the supine position. The CON, RAP, and MEL mice received only the vehicle gas. During the experiment, the vital signs of all rats were maintained within the normal range.

### Morris Water Maze (MWM) Experiment

Because we assign mice to groups according to random principles, we did not test the behavior of mice before isoflurane exposure and directly performed MWM test after isoflurane exposure. As described previously (Lugo et al., 2012), the MWM test was employed to quantify the degree of spatial learning and memory function of mice after 24 h of isoflurane exposure. The experiment was performed in a circular pool surrounded with opaque water (whitened by adding non-toxic paint). During the training, a platform measuring 12 cm in diameter and 1.5 cm thick was constant just below the surface of the circular pool. A video camera, connected to a digital tracking device (Noldus Information Technology, Leesburg, VA, United States), was used to monitor the movement of the mice. Training trials were performed four times/day for four consecutive days. The mice were released randomly in the pool and allowed 60 s to locate the platform. Following the failure to find the same, the mice were guided onto the platform, where they were kept for at least 15–20 s and then released again in the pool at a different location; the time taken to locate the platform was recorded. After 24 h of test trials, all animals were required to undergo a 60-s probe trial. In the probe trial, animals were released into the same pool albeit without the platform. The retention was determined by the time spent by the mice in the quadrant that contained the platform and the frequency at which each animal crossed the former location of the platform.

### Western Blot Analysis

On the day that the MWM experiment was completed, mice were euthanized using an overdose of pentobarbital followed by cervical dislocation. For Western blot analysis, hippocampi, resected from the brain tissue, were stored at −80°C until further use. The hippocampal specimens were homogenized in RIPA buffer consisting of 150 mM NaCl, 0.1% sodium lauryl sulfate, 0.5% sodium deoxycholate, 10% Triton X-100, 50 mM tromethamine, and protease inhibitor cocktail. Subsequently, the lysate was centrifuged for 25 min at 12,000 × *g* at 4°C, and protein concentrations of the supernatants were determined using a Bradford protein assay kit (Beyotime Institute of Biotechnology Co., Ltd., Shanghai, China). An equivalent of 30 μg sample was resolved by 8–12% sodium dodecyl sulfate-polyacrylamide gel electrophoresis (SDS-PAGE) and transferred to a polyvinylidene fluoride membrane (PVDF; EMD Millipore, Billerica, MA, United States). All membranes were blocked for 60 min with 5% skim milk in Tris-buffered saline (TBS) buffer before probing overnight at 4°C with the following primary antibodies: rabbit polyclonal anti-phosphorylated (phospho)-mTOR (cat. no. 2971, 1:500; Cell Signaling Technology, Inc., Boston, MA, United States), rabbit polyclonal anti-mTOR (cat. no. 2972, 1:500; Cell Signaling Technology), anti-β-actin (A3853, 1:2,000; Sigma, St. Louis, MO, United States). Subsequently, the membranes were rinsed in TBS-supplemented before incubation with the corresponding secondary horseradish peroxidase-conjugated goat anti-rabbit IgG antibody (Beijing Zhongshan Golden Bridge Biotechnology Co., Ltd., Beijing, China) at room temperature for 1 h. The immunoreactive bands were visualized using an enhanced chemiluminescence detection kit (EMD Millipore), and the band intensity was determined by densitometric analysis. The relative protein expression levels were normalized relative to the actin.

### Enzyme-Linked Immunosorbent Assay (ELISA)

Blood samples were withdrawn from the jugular vein catheter under deep anesthesia. Blood was centrifuged for 10 min at 2,600 × *g*, and plasma was collected and stored at −80°C until further analysis. The hippocampus tissue used the same homogenized sample for Western blots analysis. The levels of TNF-α, IL-1β, and IL-6 in the hippocampus as well as melatonin in both plasma and hippocampus were quantified using a sandwich ELISA assay according to the manufacturer’s instructions, respectively (TNF-α, IL-1β, and IL-6 ELISA kits were purchased from Multiscience, Zhejiang, China; melatonin ELISA kit was purchased from Elabscience, Wuhan, China). Both inflammatory mediators and melatonin in the hippocampus are expressed as pg/mg of tissue, whereas melatonin in plasma is described as pg/ml of tissue.

### Statistical Analysis

The GraphPad Instat 5.0 software (La Jolla, CA, United States) was used to perform all the statistical analyses. For comparison of two groups, Student’s *t* test was used. For multiple comparisons, a one-way analysis of variance (ANOVA) test was used, followed by SNK test. All data are expressed as mean ± standard error or mean ± standard deviation. *P* < 0.05 was considered statistically significant.

## Results

### Level of Melatonin in the Hippocampus and Plasma Declined After Isoflurane Exposure, and Intraperitoneal Injection of Exogenous Melatonin Reversed the Decrease in Melatonin Level in the Plasma and Hippocampus

**FIGURE 2:**
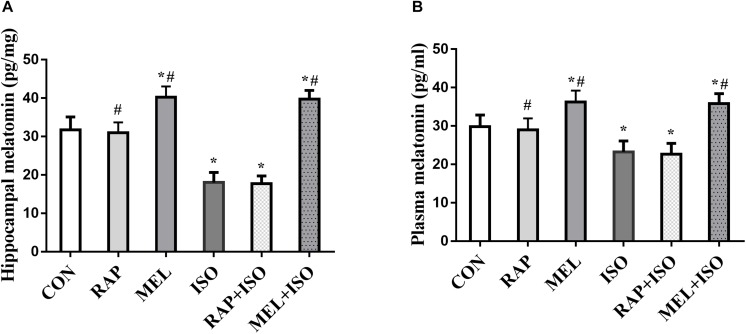
Melatonin reverses isoflurane-induced decrease in hippocampal and plasma melatonin levels. **(A,B)** Hippocampus and plasma melatonin levels were significantly decreased after isoflurane exposure, with exogenous melatonin (10 mg/kg) markedly reversing the reduction in melatonin levels that occur post-isoflurane exposure. All data are depicted in terms of mean ± SD (*n* = 12). ^∗^*P* < 0.05 versus CON group; ^#^*P* < 0.05 versus ISO group.

To determine the effect of isoflurane exposure and intraperitoneal injection of exogenous melatonin in hippocampus and plasma, ELISA was carried out upon completion of the probe MWM trial. Compared to CON, the level of melatonin in the hippocampus and plasma decreased in ISO and RAP + ISO (*P* < 0.05, Figure 2). Compared to ISO group, the level of melatonin increased in the plasma and hippocampus in MEL and MEI + ISO (*P* < 0.05, Figure 2).

### Isoflurane Exposure Impaired the Memory and Spatial Learning Abilities in Mice, Whereas Rapamycin and Melatonin Improved the Isoflurane-Induced Deficits in Memory and Spatial Learning Abilities

**FIGURE 3:**
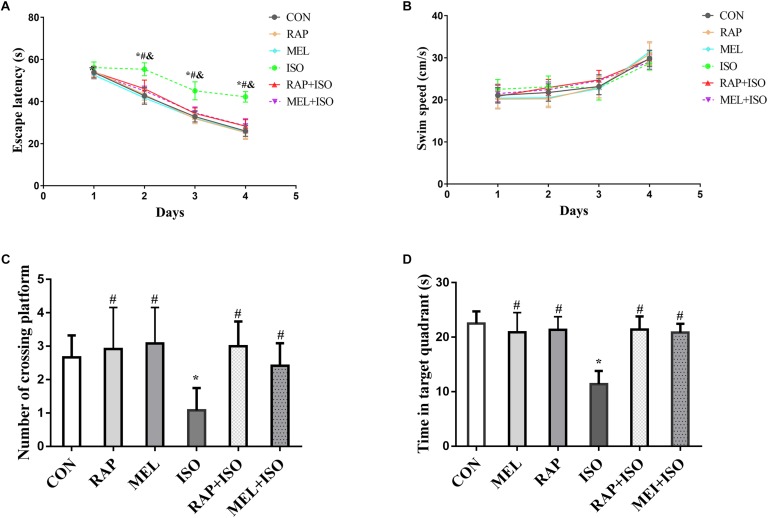
Rapamycin and melatonin suppresses isoflurane-induced decline in cognitive impairment. **(A)** Isoflurane resulted in prolonged escape latency in the Morris water maze (MWM) test. Rapamycin and melatonin both reduced the increased duration of isoflurane-induced prolongation of escape latency. Data are depicted in terms of mean ± SD (*n* = 12). ^∗^*P* < 0.05 ISO group versus CON group; ^#^*P* < 0.05 RAP + ISO group versus ISO group; ^&^*P* < 0.05 MEL + ISO group versus ISO group. **(B)** Isoflurane had no effect on mice swimming speed. **(C,D)** Isoflurane lowered the number of platform location crosses and the target quadrant dwell time, and melatonin suppressed the isoflurane-induced lowering in number of platform location crosses and target quadrant dwell time. There was no difference among the CON, RAP, RAP + ISO, and MEL + ISO groups in the result of the MWM. Data are depicted as the mean ± standard error (*n* = 12). ^∗^*P* < 0.05 versus CON group; ^#^*P* < 0.05 versus ISO group.

The MWM test was employed to determine the effect of rapamycin and melatonin on isoflurane-induced deficits in memory and spatial learning activities. The escape latency of mice in the ISO group was significantly higher than those in the CON group (*P* < 0.05, Figure 3A). Conversely, the escape latency was significantly decreased in RAP + ISO and MEI + ISO groups as compared to the mice in the ISO group. There is no difference in cognitive function between CON, RAP, MEL, RAP + ISO, MEL + ISO groups (*P* < 0.05, Figure 3A). However, swimming speed did not differ significantly among the groups (*P* > 0.05, Figure 3B). The probe test, dwelling time in the target quadrant, and the number of platform location crosses were markedly decreased in the ISO group as compared to the CON group (*P* < 0.05, Figures 3C,D). On the other hand, both of the parameters of the MWM test were significantly higher in RAP + ISO and MEI + ISO groups as compared to those in the ISO group. There is also no difference in cognitive function among the CON, RAP, MEL, RAP + ISO, and MEL + ISO groups (*P* < 0.05, Figures 3C,D).

### Isoflurane Elevated Hippocampal p-mTOR Expression Whilereas Rapamycin and Melatonin Suppressed the p-mTOR Expression Post-isoflurane Exposure

**FIGURE 4:**
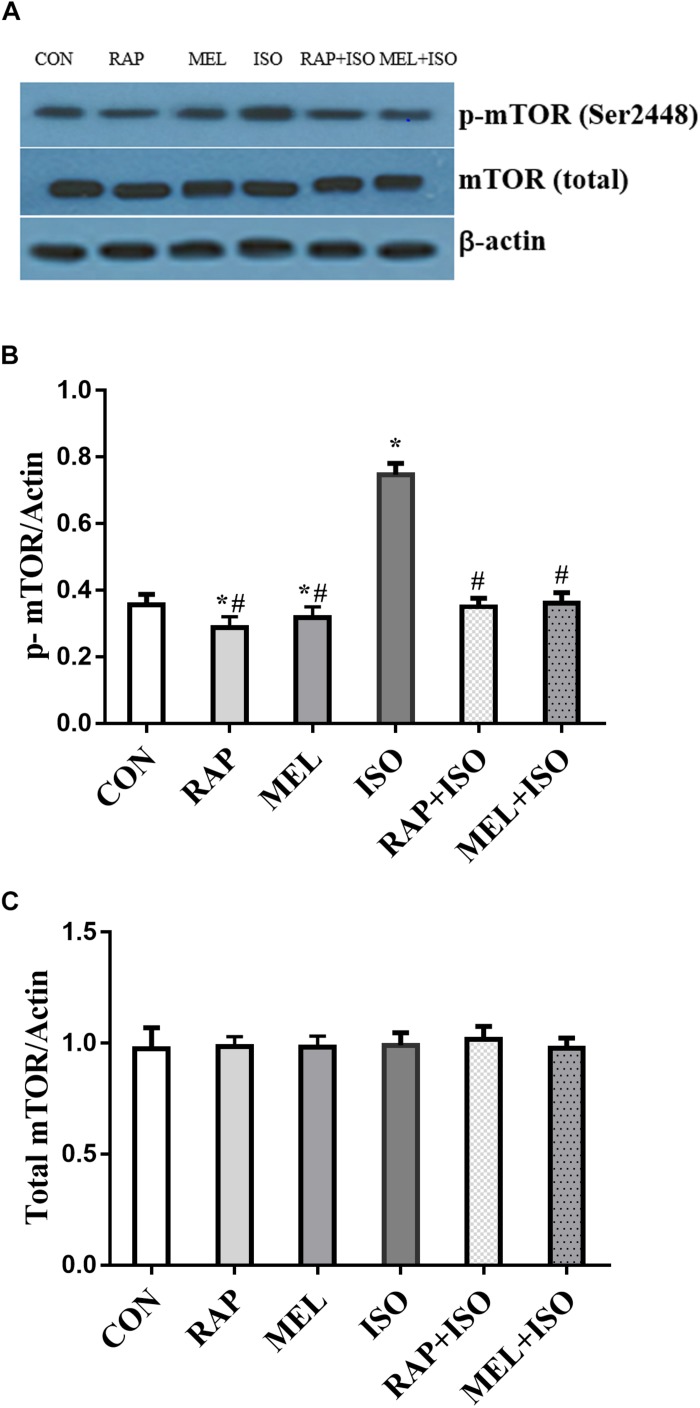
Rapamycin and melatonin attenuates isoflurane-induced hippocampal p-mTOR increase. **(A,B)** Isoflurane caused an increase in p-mTOR expression in the hippocampus. Rapamycin and melatonin suppressed the expression of p-mTOR after isoflurane exposure, and rapamycin and melatonin also reduced the expression of p-mTOR in untreated mice. **(A,C)** Total mTOR was unchanged among all of the groups. All data are depicted in terms of mean ± SD (*n* = 12). ^∗^*P* < 0.05 versus CON group; ^#^*P* < 0.05 versus ISO group.

To determine the effect of isoflurane exposure and melatonin on the expression of mTOR in the hippocampus, we examined the phosphorylation and total status of mTOR by Western blot. The expression of p-mTOR was significantly increased in the ISO group than in the CON group (*P* < 0.05, Figures 4A,B). However, the expression was significantly decreased in both RAP + ISO and MEI + ISO groups as compared to the ISO group, and the expression was also reduced in RAP and MEL groups (*P* < 0.05, Figures 4A,B). Moreover, the total mTOR expression did not differ significantly among the groups (*P* > 0.05, Figures 4A,C).

### Isoflurane Enhanced Hippocampal Inflammatory Response, Whereas Rapamycin and Melatonin Attenuated the Inflammatory Response After Isoflurane Exposure

**FIGURE 5:**
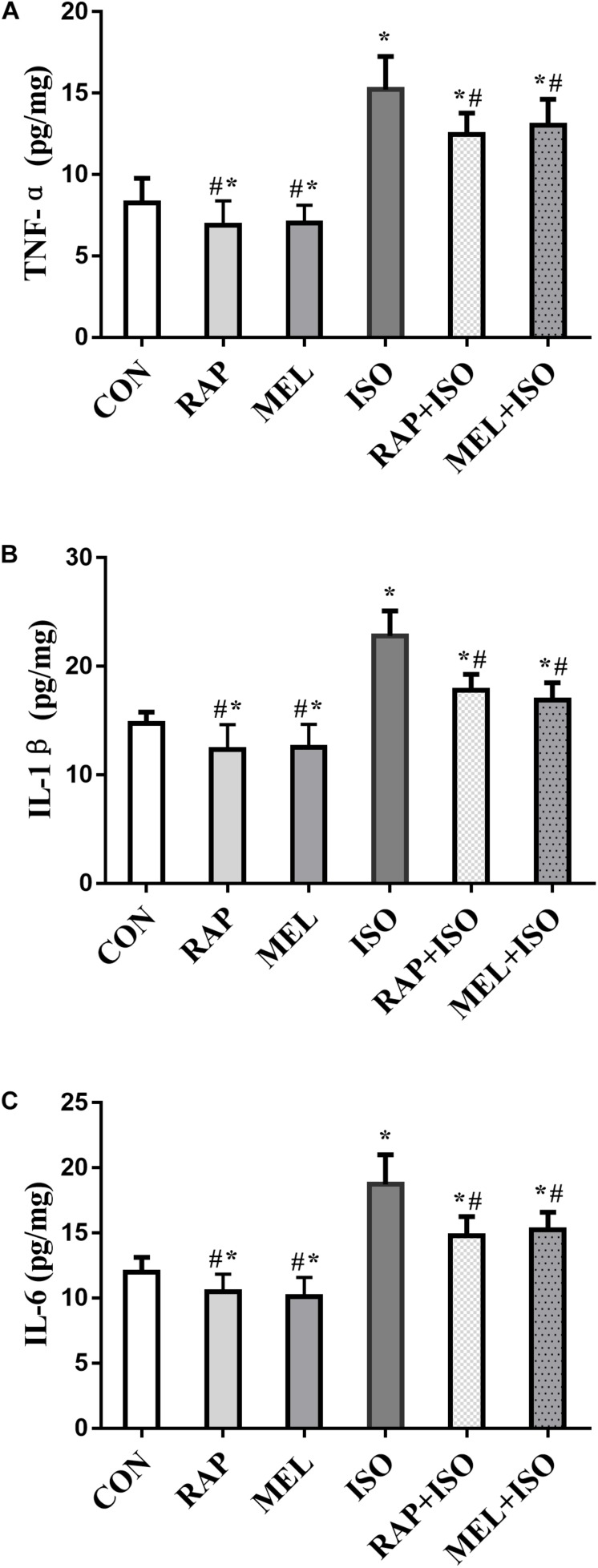
Rapamycin and melatonin attenuates isoflurane-induced hippocampal inflammation. **(A)** Rapamycin and melatonin attenuated the increase of tumor necrosis factor (TNF)-α level in the hippocampus after isoflurane exposure and untreated. **(B)** Rapamycin and melatonin attenuated the increase of interleukin (IL)-1β level in the hippocampus after isoflurane exposure and untreated. **(C)** Rapamycin and melatonin attenuated the increase of IL-6 level in the hippocampus after isoflurane exposure and untreated. All data are depicted as the mean ± SD (*n* = 12). ^∗^*P* < 0.05 versus CON group; ^#^*P* < 0.05 versus ISO group.

To determine the level of inflammatory factors in the hippocampus, we examined the concentration of TNF-α, IL-1β, and IL-6 by ELISA. The expressions of these cytokines in mice belonging to the ISO group were significantly increased as compared to those in the CON group (*P* < 0.05, Figures 5A–C), whereas the levels were markedly lower in both RAP + ISO and MEI + ISO groups than those in the ISO group, and the inflammation in the RAP and MEL groups was also reduced relative to the CON group (*P* < 0.05, Figures 5A–C).

## Discussion

These investigations supported that 4-h exposure to 2% isoflurane induces cognitive dysfunction while decreasing the levels of melatonin in the hippocampus in aged mice. mTOR inhibitor of rapamycin and exogenous melatonin exerted protective effects against cognitive dysfunction as well as against changes in mTOR and the expression profiles of inflammatory factors that are induced by isoflurane.

Whereas isoflurane is commonly employed as an inhaled anesthetic, several reports have emerged alluding to its detrimental effects on the cognitive functions in the rodent (Lin et al., 2012; Li et al., 2014). Neuroinflammation is a significant contributor to postanesthesia and postsurgery cognitive impairment (Terrando et al., 2011), and the pathobiology that dictates the effects of anesthesia on neuroinflammation is under intensive focus. Ni et al. (2013) demonstrated that the rodents exposed to 2% isoflurane for 4 h exhibited impaired cognitive functions; therefore, we employed the isoflurane treatment protocol to establish the PND model. Another study showed that isoflurane-induced cognitive dysfunction was mediated by IL-1β (Cao et al., 2012). Also, IL-1β mediates an increase in the cerebral expression of IL-6 and TNF-α, especially after isoflurane exposure (Wu et al., 2012). Furthermore, the current study demonstrated that the hippocampal expressions of TNF-α, IL-1β, and IL-6 are raised in aged mice that received isoflurane exposure, which was in agreement with previous studies. In addition, the aged mice showed memory and learning dysfunction after isoflurane exposure. These findings emphasized that hippocampal inflammatory response might be the mechanism for cognitive impairment after isoflurane exposure.

Mammalian target of rapamycin represents a serine/threonine protein kinase that is highly conserved and controls a series of cell growth activities including cell energy metabolism. Moreover, a strong association exists between the proinflammatory signaling pathways and mTOR, reinforcing the role of mTOR in dictating the outcome of inflammatory diseases. The prevention of cognitive damage induced by pathological damage has been shown to be prevented by an mTOR allosteric inhibitor, rapamycin. As a result, the *N*-methyl-D-aspartate signaling pathway was enhanced, whereas the IL-1β levels were reduced; both are responsible for ameliorating the cognitive defects that occur during the process of normal aging (Majumder et al., 2012).

Further studies demonstrated that aldosterone-triggered TNF-α expression could be inhibited by administering rapamycin to cultured tubular cells (Wang et al., 2015). Okamoto et al. (2016) concluded that NF-κB induces mTOR activation, which in turn worsens the retinal inflammatory responses. The study also demonstrated that the levels of inflammatory cytokines, such as IL-6 and monocyte chemoattractant protein-1 (MCP-1), were lowered when treated with rapamycin (Okamoto et al., 2016). Moreover, it has also been suggested that the nervous system activity is dictated partially through mTOR signaling (Maiese, 2016). A study in a cerebral palsy mouse model proved that rapamycin could decrease the neuroinflammation that was induced by hypoxia-ischemia and lipopolysaccharide (Srivastava et al., 2016). In addition, rapamycin can significantly inhibit the inflammation by inhibiting the mTOR signaling that enhances the anti-inflammatory activity of regulatory T cells (Tregs), thereby inhibiting microglia and macrophage-mediated proinflammatory cytokines and chemokines in ischemic stroke rats (Xie et al., 2014). In the present study, rapamycin caused a marked improvement despite memory impairment induced by isoflurane exposure, which suggested that mTOR signaling was involved in postoperative cognitive deficits. Furthermore, rapamycin also reduced the expression of inflammatory factors in the hippocampus, indicating that mTOR might aggravate the hippocampal neuroinflammation to participate in the pathophysiology of PND.

Melatonin is a potent antioxidant with no side effects even in the case of large-scale use. The neural injury models, such as cognitive impairment, exhibited neuroprotective effects (Liu Y. et al., 2013; Liu et al., 2017). Our experimental results also showed that melatonin reduces the cognitive impairment induced by isoflurane exposure. Currently, melatonin is under intensive focus owing to its potential anti-inflammatory effect. Melatonin administration was effective in upregulating the STAT1 DNA-binding activity, thereby suppressing the levels of proinflammatory cytokines, such as NOS and IL-6β (Tsai et al., 2011). Ding et al. (2014) found that melatonin significantly decreased the release of proinflammatory cytokines via inactivation of the mTOR pathway in the traumatic brain injury model. However, whether melatonin reduces the hippocampal inflammation in the PND model has not yet been explored. In the current study, we found that melatonin markedly decreased the release of hippocampal proinflammatory cytokines induced by isoflurane exposure. Furthermore, melatonin administration significantly inhibited the mTOR pathway. Thus, the possible mechanism for melatonin might be alleviating the cognitive impairment induced by isoflurane exposure, which downregulates the proinflammatory cytokines by inhibiting the mTOR pathway in the hippocampus.

The aged animals are likely to suffer cognitive dysfunction postoperatively, and hence, aged mice were selected to establish the PND model. Each aged animal might have different degrees of cognitive and movement deteriorations, which might affect the MWM result. Moreover, the MWM test was not conducted before the drug treatment to confirm the difference in cognition and movement deteriorations between the six groups of aged mice to exclude the potential experimental bias, which is the limitation of the current study.

## Conclusion

The present study alleviated the existing knowledge of the mechanism of the protective effects of melatonin on the brain. Our experiments revealed that the inactivation of the mTOR signaling pathways through melatonin might exert neuroprotective functions in aged mice with cognitive dysfunction secondary to isoflurane induction. This protective effect is supported by the decreased level of proinflammatory cytokines in the hippocampus. Nevertheless, additional studies would fully elucidate the mechanisms through which melatonin deactivates mTOR.

## Data Availability Statement

The datasets generated for this study are available on request to the corresponding author.

## Ethics Statement

The animal study was reviewed and approved by Ningbo University Laboratory Animal Center.

## Author Contributions

HY and JC conceived and designed the experiments. HY, GW, and XZ performed the experiments. HY, BL, and BM analyzed the experimental data. HY, JC, and GW wrote the manuscript.

## Conflict of Interest

The authors declare that the research was conducted in the absence of any commercial or financial relationships that could be construed as a potential conflict of interest.
